# Measuring research impact: a large cancer research funding programme in Australia

**DOI:** 10.1186/s12961-018-0311-3

**Published:** 2018-05-09

**Authors:** Jacqueline A. Bowden, Nicole Sargent, Steve Wesselingh, Lincoln Size, Claire Donovan, Caroline L. Miller

**Affiliations:** 1 South Australian Health and Medical Research Institute, Adelaide, Australia; 2 Cancer Council South Australia, Adelaide, Australia; 3 University of Adelaide, School of Public Health, Adelaide, Australia; 4 Division of Health Sciences, Brunel University London, Uxbridge, UK

**Keywords:** cancer, Payback Framework, impact, research

## Abstract

**Background:**

Measuring research impact is of critical interest to philanthropic and government funding agencies interested in ensuring that the research they fund is both scientifically excellent and has meaningful impact into health and other outcomes. The *Beat Cancer Project* (BCP) is a AUD $34 m cancer research funding scheme that commenced in 2011. It was initiated by an Australian charity (Cancer Council SA), and supported by the South Australian Government and the state’s major universities.

**Methods:**

This study applied Buxton and Hanney’s Payback Framework to assess research impact generated from the BCP after 3 years of funding. Data sources were an audit of peer-reviewed publications from January 2011 to September 2014 from Web of Knowledge and a self-report survey of investigators awarded BCP research funding during its first 3 years of implementation (2011–2013). Of the 104 surveys, 92 (88%) were completed.

**Results:**

The BCP performed well across all five categories of the Payback Framework. In terms of knowledge production, 1257 peer-reviewed publications were generated and the mean impact factor of publishing journals increased annually. There were many benefits to future research with 21 respondents (23%) reporting career advancement, and 110 higher degrees obtained or expected (including 84 PhDs). Overall, 52% of funded projects generated tools for future research. The funded research attracted substantial further income yielding a very high rate of leverage. For every AUD $1 that the cancer charity invested, the BCP gained an additional AUD $6.06. Five projects (5%) had informed policy and 5 (5%) informed product development, with an additional 31 (34%) and 35 (38%) projects, respectively, anticipating doing so. In terms of health and sector and broader economic benefits, 8 (9%) projects had influenced practice or behaviour of health staff and 32 (34%) would reportedly to do so in the future.

**Conclusions:**

Research impact was a priority of charity and government funders and led to a deliberate funding strategy. Emphasising research impact while maintaining rigorous, competitive processes can achieve the joint objectives of excellence in research, yielding good research impact and a high rate of leverage for philanthropic and public investment, as indicated by these early results.

**Electronic supplementary material:**

The online version of this article (10.1186/s12961-018-0311-3) contains supplementary material, which is available to authorized users.

## Background

Cancer death rates have continued to fall in Australia since the 1980s, with incidence rates also decreasing. During this time, survival rates have improved substantially and some of these gains can be attributed to improvements in detection and developments in treatment through research and new technologies [1]. According to WHO, people living in Australia generally have better cancer survival than those living in other countries and regions [2]. However, there is much work yet to be done, with more cancer cases being diagnosed each year in Australia [1].

Translational research has been an important contributor to reduced cancer incidence and increased survival. However, the process of translating research into policy and practice is often convoluted and slow. It is commonly stated that it takes an average of 17 years for research evidence to be translated into clinical practice [3–5]. With growing competition for the fundraising dollar, charities are under increasing pressure to demonstrate and report the impact of their work to the community, and governments are seeking to allocate scarce resources effectively. As a result, researchers are increasingly being asked to demonstrate the impact of their research in terms of improved treatments and health gains to inform funding decisions [6].

Measuring the impact of research is an important area, and one that is relatively underdeveloped. In 2001, Smith noted, “*The main aim of health research is to improve the health of people. Yet the performance of researchers tends to be measured by the scientific quality of their research rather than by its impact on health*” [7]. Academic research metrics are focussed on peer-review publications, journal quality and prestige, journal impact factors, and article citations. While these measures are important, particularly to measuring impact within the scientific community, they do not measure impact in terms of translation into practice and policy, or return on investment and rate of leverage for charities and their donors. The need to capture, measure and monitor research impact more broadly is receiving increasing attention in Australia and internationally [8–10].

Cancer Council SA is one of the largest cancer research funding bodies in South Australia. Researchers go through rigorous, competitive processes to obtain funding, which ensures that the research funded is of the highest quality. Like many philanthropic agencies, Cancer Council SA became increasingly interested in being able to capture and demonstrate to the community the impact of the research that it was funding in terms of cancer control. Within this context, they initiated a review of the impact of the research they fund.

Cancer Council SA’s *Beat Cancer Project* (BCP) is an AUD $34 m, 10-year competitive funding scheme for cancer research in South Australia, funding over 100 cancer research initiatives in its first 3 years. Cancer Council initiated the BCP with an AUD $10 m investment, matched by the South Australian Government (via the Department for Health and Ageing) over 5 years. Cancer Council then added a further AUD $7 m for the next 5 years, which was matched by the South Australian Government. BCP is underpinned by a strategic cancer research partnership with the South Australian Health and Medical Research Institute and the state’s three major universities (University of Adelaide, Flinders University and the University of South Australia). These universities provide additional matched funding (along with other organisations) for the majority of schemes within the competitively awarded research.

A widely accepted framework for evaluating the impact of research is the Payback Framework [11–16], developed by Buxton and Hanney (Brunel University London, United Kingdom) to examine the impact or ‘payback’ of health services research [17]. The Payback Framework consists of two elements – a logic model of the research processes and the five categories of ‘paybacks’ or impact that may be gained from research [17], namely (1) knowledge production; (2) benefits to future research and research use; (3) benefits to informing policy and product development; (4) health and sector benefits; and (5) broader economic benefits. In Australia, Donovan et al. [16] used the Framework to evaluate the payback profiles of the National Breast Cancer Foundation’s funded research over a 17-year period. The study found that 46% of survey respondents reported career progression, and 185 higher degrees were either obtained or expected, including 121 PhDs. Overall, 66% produced tools that built capacity across the research system and research teams leveraged $1.40 in funding for every $1 invested [16]. The Payback Framework has been used in other fields of research, including cardiovascular research [6], arthritis research [11] and asthma research [15]. However, the Payback Framework has not yet been applied in a general cancer setting.

This study aims to assess the impact of a cancer research funding programme, expanding beyond traditional research metrics. This review applied the Payback Framework to the BCP, which at the time of this review, was in its third year. This is the first in-depth study of the impact of a general cancer research programme using the Payback Framework.

## Methods

### Records held by Cancer Council SA’s BCP

Administrative records were obtained for funded research, including amounts of grant funding awarded. BCP grants during 2011–2013 included infrastructure grants to support clinical trials (ongoing); data linkage (ongoing); data collection systems and equipment (one-off grants); research project grants (duration 1 year); blue sky funding (1 year); partnership grants (3 years); hospital research packages (up to 5 years) and workforce funding, including research chairs/leaders (5 years); principal research fellowships (4 years); fellowships (3 years); PhD top-up scholarships (one-off grants); and travel grants (one-off grants). The distribution of funding during the study period (2011–2013) was 58.9% for biomedical research, 21.6% for population/health services research and 19.5% for clinical research and across the cancer spectrum.

### Publications audit

To assess knowledge production, a separate audit was undertaken of all peer-reviewed papers published within the period of January 1, 2011, and September 5, 2014, by chief investigators using Web of Knowledge. The audit was undertaken separately to the survey of chief investigators to reduce respondent burden and to reduce the potential negative impact on response rates. The journals published in most frequently were tabulated to construct a list of the top 20.

### Survey of chief investigators

A survey tool was developed using the key concepts from the Payback Framework and its five categories of impact. The survey was also consistent with the National Breast Cancer Foundation (NBCF) survey (14). A full list of questions is included in Additional file 1.

The survey was emailed to 104 BCP recipients in June 2014 (six recipients had multiple funding on similar projects so they were asked to complete one survey only). The response rate for the surveys was 88% (92/104). Data were analysed using SPSS version 22.

## Results

### Knowledge production

#### Publications

In total, 1257 peer-reviewed publications were generated by BCP recipients over the 3-year period, yielding an average of 11.3 per grant awarded. The impact factor for the journals in which BCP researchers published most frequently increased annually, from 3.84 in 2011 to 6.74 in 2013, indicating that, over time, work is being published in higher impact journals. The overall impact factor for the journals in which BCP recipients most commonly published in over the period was 6.40. Table 1 presents the 20 peer-reviewed journals most commonly published in for the study duration. Table 2 outlines the most commonly cited article for the three funding categories (biomedical, clinical and population health/services).

**Table 1:** Top 20 peer-reviewed journals most commonly published in for 2011–2013

Journal	Number of publications	Impact factor
		(Web of Knowledge 2012)
*Blood*	60	9.78
*Journal of Clinical Oncology*	48	18.04
*Asia Pacific Journal of Clinical Oncology*	47	0.91
*PLoS One*	18	3.73
*Leukemia*	17	10.16
*Medical Journal of Australia*	15	2.85
*Supportive Cancer Care*	14	3.09
*Gastroenterology*	12	12.82
*Journal of Gastroenterology and Hepatology*	12	3.33
*Cell Death and Differentiation*	11	8.24
*Oncogene*	11	7.36
*ANZ Journal of Surgery*	10	1.50
*International Journal of Cancer*	10	6.20
*Oncotarget*	10	6.64
*Haematologica*	9	5.94
*Cancer Biology and Therapy*	9	3.29
*British Journal of Haematology*	8	4.94
*Journal of Clinical Investigation*	8	12.81
*British Journal of Cancer*	8	5.08
*Asian Pacific Journal of Cancer Prevention*	8	1.27
Average Total	N/A	6.40

**Table 2:** Most commonly cited article for the three funding categories

Research category	Paper	Citations (Google scholar 2013)
Clinical	Falchook G, Long G, Kurzrock R, Kim K, Arkenau T, Brown M, et al. Dabrafenib in patients with melanoma, untreated brain metastases, and other solid tumours: a phase 1 dose-escalation trial. *Lancet*. 2012;379(9829):1893–901.	136
Biomedical	Chau N, Mackenzie P, Miners J. The contribution of human udp-glucuronosyltransferase enzymes to the glucosidation of mycophenolic acid. *Drug Metab Rev*. 2014;45:S1.	129
Population/health services	Hutchinson A, Wilson C. Improving nutrition and physical activity in the workplace: a meta-analysis of intervention studies. *Health Promot Int*. 2012;27:2.	14

#### Dissemination

BCP recipients disseminated knowledge produced outside of peer-reviewed publications. Aside from publication, the most popular methods of dissemination were oral presentations, posters, conferences and workshops for academics (*n* = 590), overall representing 6.4 presentations/posters per survey grant respondent. This was followed by general public presentations (*n* = 75; 0.82 per grant), newspaper articles (*n* = 36; 0.39 per grant), radio interviews (*n* = 29; 0.32 per grant) and television interviews (*n* = 19; 0.21) per grant.

### Benefits to future research and research use

#### Research training and career development

Funded researchers reported that 110 higher degrees (1.2 per grant) were awarded or expected in the next 5 years, including 84 PhDs as a direct consequence of BCP funding. In addition, 21 (22.8%) reported that participation in the research led to career advancement for members of the funded research team (e.g. an advancement from Senior Lecturer to Professor).

#### Capacity-building

BCP recipients reported that 48 funded projects (52.2%) had generated tools (including improved websites, questionnaires and registries to procedures, methods and markers for early detection) for future research as a result of the funded research that would help to build capacity across the research system.

### Attracting further income and generating further research

#### Further income

The BCP’s investment of AUD $10.4 m during the study period yielded further funding to the amount of AUD $26.3 m (AUD $12.5 m in matched funding from universities and an additional AUD $13.8 m in further research funding from other sources, e.g. NHMRC). Thus, for every AUD $1 invested by the BCP ($0.50 by SA Health and $0.50 by Cancer Council SA), research teams gained an additional $2.53. Or in other funding leverage terms, for the AUD $5.2 m invested by Cancer Council SA, a further AUD $31.5 m was achieved in funding from other sources (SA Health, Universities, NHMRC, etc.), meaning that, for every Australian dollar invested by Cancer Council SA on behalf of their donors, the BCP gained an additional AUD $6.06.

#### Further research

Overall, 38 (41.3%) of BCP investigators reported that their funded research findings, methodology or theoretical developments generated subsequent research. In addition, 16 investigators reported that their research contributed to research conducted by others.

### Benefits from informing policy and product development

#### Policy development

**Fig. 1:**
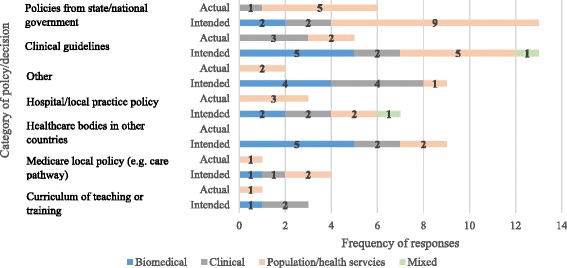
Intended and actual use in informing policy development/decision-making by main research type (frequency)

Impact into policy and practice had already occurred in some instances, despite the short 3-year time frame, but was most frequently intended for the future. The survey found that five BCP recipients reported that their results had been used in policy and decision-making and a further 31 (34%) reported that they plan to do so in the future, but that the timeframe since BCP funding has been insufficient to have done so yet. Actual use ranged from impact on refined treatment guidelines for colorectal cancer, high-risk patients, liver transplant and liver resection patients, the management of women with early breast cancer (including stratification of women according to their risk of subsequent breast cancer), physical activity for cancer survivors, to promoting case conferencing for palliative care, increasing expenditure on anti-smoking mass media, and informing position statements for the non-government sector. Expected use ranged from use in state and national policy in tobacco control and obesity, to guidance on improvements for the health of Aboriginal and Torres Strait Islander communities, and clinical management of women with screen-detected breast lesions. Population health/health services researchers listed 35 ways of expected/actual use, whereas biomedical researchers listed 20 and clinical researchers listed 19. There were differences in the levels at which policies were, or were likely to be, influenced by research type (Fig. 1). The impact (or intended impact) of population and health services research was most often in government policies and hospital/local practice and policy. Biomedical research was reported as being most likely to impact clinical guidelines and healthcare bodies in other countries. Clinical research was reported to be most likely to impact clinical guidelines, and curriculum or training.

In total, 58% of projects reported that they had interaction with end-users of their research, namely policy-makers, practitioners and/or consumers, either before (39.1%), during (43.5%) or after project completion (34.8%). Further analysis revealed that a higher proportion of population/health services (71%) and clinical researchers (67%) had engagement than biomedical researchers (54%).

#### Product development

Five (5%) BCP recipients reported that they had used their results to inform product development (including pharmaceuticals, diagnostic tests, medical devices, etc.) and a further 35 (38%) expected their research to do so in the future.

### Health gains and broader economic benefits

**Fig. 2:**
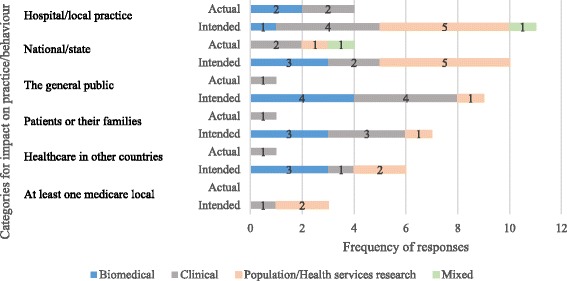
Actual and intended impacts to practice and behaviour (frequency)

Survey results found that 8 (9%) BCP recipients reported influencing practice or behaviour of health service staff, patients and the public, and a further 32 (35%) reported that their projects would do so in the future. Actual and expected benefits ranged from decreasing the side-effects of cancer treatments to prevention and early detection. The majority of participants reported that they had increased, or expected to do so, the length or quality of life for people with cancer (57%). There were differences in the levels at which policies were, or were likely to be, influenced by research type (Fig. 2).

## Discussion

With more cancer cases being diagnosed each year in Australia, there is a responsibility to ensure that the research conducted is of high impact. This study is the first of its kind attempting to capture research impact in a general cancer research setting. The NBCF study used a similar methodology for their research investment, and therefore some basic comparisons can be made. However, it is important to note that Cancer Council SA’s BCP had only been funded for 3 years at the time of the survey, compared with the NBCF study that evaluated funding over a 17-year period. The high response rate for this survey means that the conclusions are representative of the whole BCP portfolio.

The BCP had outstanding performance in generating further research and funding, an important outcome for the funders and donors. Due to its unique and deliberate funding strategy, the BCP demonstrated a very high rate of leverage for Cancer Council SA donors, whereby, for every AUD $1 donated, the BCP gained an additional AUD $6.06 in research funding. In terms of return on investment, for every AUD $1 spent by the BCP, research teams gained an additional AUD $2.53. This compares very favourably to the NBCF investment (which had a different strategy and may not have required universities to co-invest) where, for every AUD $1 spent, an additional AUD $1.40 was leveraged [16]. This result highlights the potential of charities working with governments and other funding bodies to achieve a high return on investment and rate of leverage for donors.

The BCP had slightly less impact than the NBCF in terms of knowledge production. Research dissemination in the form of conference presentations appeared to be slightly lower for BCP recipients (6.4 per grant) compared to the NBCF-funded research (8.0 per grant). Interviews with the media were also lower in BCP participants (0.45 per grant) compared to NBCF grant recipients (1.1 articles per grant). These results most likely reflect the longevity of the NBCF funding programme and the experience its funded researchers have with NBCF’s expectations in this regard.

Overall, the two Australian cancer research funding programmes were comparable in engagement with end-users, consumers, clinicians and policy-makers (58% in BCP and 56% in NBCF), as well as in research training, with BCP resulting in 110 higher degrees obtained or expected (1.2 per grant) compared with 185 by NBCF (1.2 per grant). Career development took place in 23% of BCP team members (over a 3-year period) compared with 50% in the NBCF (over a 17-year period).

Importantly, this study found that, by purposefully funding research across the spectrum (biomedical, clinical and population/health services research), there was diversity in the impact on policy, practice and behaviour, health gain and broader economic impacts. For example, population health research was more likely to impact on government health policy, whereas clinical practice, training and curriculum were more likely to be impacted by clinical research, which is likely to yield greater outcomes for the community and people affected by cancer. The results of this study have been incorporated into a review of the scheme and its renewal for a further 5 years. The results provide further substantiation to the funders’ strategy of funding across all three streams, and have led to a marginal adjustment of funding ratios. Impact evaluation will also be embedded in future surveys and another in-depth study will be undertaken in 5 years with these recipients to determine whether the intended impacts have indeed come to fruition.

### Limitations

It is important to note that caution must be applied when interpreting the results as there has been a very short-time frame between the commencement of this project and the survey. The often cited time-lag in translation of research is 17 years [5]; thus, with this in mind, the findings of this study should be viewed as early impact. In addition, the audit of peer-reviewed publications is merely indicative and further bibliometric analysis by funding type would be beneficial as would analysis by alternative traditional metrics such as citations (or other measures) rather than relying on impact factors. Impact factors are now widely criticised as a flawed indicator for the quality of each individual paper within that journal [18]. Furthermore, the methods of actual translation and impact factors for population health and health services journals are often quite different to the impact factors for clinical and biomedical journals and are therefore not directly comparable. Responses within the survey are largely self-reported, with the inherent biases that self-reporting brings. In addition, in order to increase response rates, chief investigators were asked to complete the report as a condition of funding and were advised that a failure to submit the report may render them ineligible for future funding. This is likely to have increased response rates but may also impact on responder bias. While reports of actual impact can be verified, projections of future impact are much harder to check for likelihood. The BCP has been funded for a second 5-year term and a further impact assessment is recommended, allowing more accurate assessment of actual impact as well as a comparison of projected and actual impact.

## Conclusion

This is the first study of its kind using the Payback Framework in a general cancer research setting. Despite the short time frame, results are favourable and highlight the potential importance of setting impact evaluation in place at the commencement of funding to influence expenditure around research impact. The BCP compared well with a previous study on all categories and compared particularly well in generating further funds to build upon the research (for every $1 spent by the BCP, a further $2.53 were generated by research teams). This survey highlights the potential importance of funding research in all areas (biomedical, clinical and population health/health services research) to achieve and maximise payback on all of the categories. This may serve to maximise impact of research, which is likely to translate to broader impacts for the community. Ultimately, with translational research being funded across the spectrum, there is a likelihood that more cancers will be prevented, rates for people with cancer may be increased, and the journey for those affected by cancer is likely to be improved. This study can provide a benchmark for other research in the general cancer setting and for future assessments of the BCP. The results of this study are timely in the context of increasing pressure by governments and charities to justify the impact of their funded research more broadly.

## Additional file

Additional file 1: Appendix 1 Chief investigator survey questions. (DOCX 14 kb)

## Abbreviations

BCP: *Beat Cancer Project*
NBCF: National Breast Cancer Foundation
