# Supplementary material for: Measuring research impact: a large cancer research funding programme in Australia
Source: Health Res Policy Syst. 2018 May 9;16:39. doi: 10.1186/s12961-018-0311-3 (PMC5944042; doi:10.1186/s12961-018-0311-3)
Supplement: Supplementary file 1 — Appendix 1 Chief investigator survey questions. (DOCX 14 kb) [file 12961_2018_311_MOESM1_ESM.docx]

**Appendix 1: Chief investigator survey questions**

*Dissemination:* ‘Indicate the number of each of the following dissemination activities that have been associated with this funded research: oral presentations; poster conferences; conferences/workshops for academics; newspaper releases; magazine releases; radio interviews/presentations; television interviews/presentations’.

*Research training and capacity building: ‘*Has or will participation in the funded research lead to any members of the project team achieving (or being likely to achieve) any formal qualifications in the next 5 years? If yes, please list’. ‘Has participation in the research led directly to career advancement for any members of the funded research team? If yes, please list’. ‘Please describe any tools for future research generated by the project and indicate if they have been used. Examples include a new or significantly improved: animal model, cell line, physiological or biochemical marker, assay, model of disease, physiological outcome measure, economic model, validated patient questionnaire etc’.

*Further income: ‘*Have the project findings, methodology or theoretical developments generated subsequent research led by members of the original project team? If yes, please give details including the funding amount’.

*Further research:* ‘Are you aware of any significant ways in which your funded research has contributed to further research conducted by others? If yes, please give details’.

*Policy development:* ‘Funded research findings can be used for a wide range of policy/decision making at any level of the health service. This includes influencing the policies of local healthcare units, trusts, national governmental bodies, professional bodies at a local or national level, health-care bodies in other countries, and also influencing the curriculum of teaching or training courses. Have the findings from your funded research already been used in any such ways e.g. being cited in a clinical guideline? Please give details’. ‘Are there any reasons for expecting the findings to be used for future policy/decision making? Please give details’. ‘Please categorise the levels at which policies/decisions were influenced: national/state; hospital/local practice; clinical guidelines, healthcare bodies in other countries, medicare local policy (e.g. care pathway) and curriculum of teaching or training’. ‘Was interaction with potential users (policy makers, practitioners, patients etc) a factor in actual or future research utilisation? Interaction before starting? Interaction during the project? Interaction after completing the project?’

*Product development:* ‘Funded research findings can be used in the development of various types of products including pharmaceuticals, diagnostic tests, medical devices etc. Have the findings from your funded research been used in any such ways? Yes/no’. ‘Are there any reasons for expecting the findings to be used in future product development? Please give details’.

*Health gains and broader economic developments:* ‘Do you expect health/health service/economic benefits arising from the applications of your funded research findings or uptake of products to be evident in the future? Please give details’. ‘The findings from research can influence the practice (including confirmed current practice) or behaviour of health service staff or patients/public, either directly or through the application of research-informed policies. Have the findings from your project already influenced practice or behaviour?’ ‘Are there any reasons for expecting the findings to influence the future practice or behaviour of health service staff or patients?’ ‘Please categorise the levels at which policies/decisions were influenced’: ‘national/state’; ‘hospital/local practice’; ‘the general public’; ‘patients or their families’; ‘healthcare in other countries’; ‘at least one medicare local’. ‘Various possible health/health service benefits can arise from the application of research findings and the uptake of new products. These benefits include: improved health; improved service delivery; cost savings; an increase in values considered desirable e.g. equity’ Have any such benefits already arisen as the result of the application of your research project findings?’.
